# High-altitude cerebral oxygen saturation detection using wireless wearable cerebral oximeter

**DOI:** 10.3389/fneur.2024.1445563

**Published:** 2024-09-30

**Authors:** Juanning Si, Yifang He, Junyuan Yao, Jian Yu, Rixing Jing, Qing He, Xin Zhang, Lijun Xiao

**Affiliations:** ^1^School of Instrumentation Science and Opto-Electronics Engineering, Beijing Information Science and Technology University, Beijing, China; ^2^Department of Psychology, Shandong Second Medical University, Weifang, China; ^3^Brainnetome Center, Institute of Automation, Chinese Academy of Sciences, Beijing, China; ^4^National Laboratory of Pattern Recognition, Institute of Automation, Chinese Academy of Sciences, Beijing, China; ^5^Department of Medical Psychology, The Third Medical Center of Chinese PLA General Hospital, Beijing, China; ^6^Faculty of Pediatrics, The Seventh Medical Center of Chinese PLA General Hospital, Beijing, China

**Keywords:** high altitude, hypoxia, cerebral oxygen saturation, cerebral oximeter, cycling

## Abstract

**Background:**

Hypobaric hypoxic conditions encountered at high altitudes can significantly impact the physiological functions of human body. Therefore, accurate and real-time monitoring of physiological characteristics is crucial for the prevention of brain injuries in individuals with acute and chronic high-altitude exposure.

**Methods:**

In this study, a wireless wearable cerebral oximeter (WORTH band) was used for the continuous, real-time monitoring of physiological parameters, including regional cerebral oxygen saturation (rSO2) and heart rate (HR), among subjects with high-altitude exposure.

**Results:**

During the high-altitude (from 46 m to 4300 m) expedition task, there was a significant decrease in rSO2 accompanied by a corresponding increase in heart rate as the altitude increased. Additionally, during the long-term (52 days) high-altitude (from 356 m to 4658 m) cycling task, the altitudes were significantly correlated with the rSO2 and SpO2 in the elderly subjects.

**Conclusion:**

The current findings indicate that the WORTH band oximeter can serve as a promising instrument for measuring rSO2 at high altitudes. We hope that the insights derived from this study could contribute to the management of cerebral oxygenation for individuals with high-altitude exposure and further expand the existing understanding of brain functional detection at high altitudes.

## 1 Introduction

Oxygen is indispensable for cellular metabolism in the human body (1). Although the brain accounting for only 2% of total body weight, it receives approximately 15–20% of cardiac output and consumes nearly 20% of the body’s total oxygen to maintain its normal functions (2). Brain metabolism absolutely relies on oxygen and glucose; however, it has a limited capacity for energy storage, necessitating a continuous energy supply through blood circulation (2). Cerebral ischemia or anoxia can lead to cognitive impairments, irreversible brain injury, and even brain death under severe circumstances. Therefore, the real-time monitoring of the oxygenation state of the brain is crucial, not only for exploring changes in cerebral hemodynamics and other physiological characteristics but also for improving clinical practices in high-altitude research (3).

High-altitude areas are often characterized by a variety of physical and physiological challenges, mainly including reduced humidity and temperature, increased exposure to ultraviolet radiation, and the presence of hypobaric hypoxic environments (1, 4). These conditions can exert substantial impacts on the physiological functions of human body (1) and produce a unique challenge to cerebrovascular system of the human brain (5). Accurate and real-time detection of rSO2 is particularly crucial for the protection of the brain in lowlanders with acute high altitude exposure (6). The hypobaric and hypoxic characteristics of high-altitude environment results in a reduction in the availability of oxygen. As altitude increases, air density and atmospheric pressure decrease correspondingly. It has been reported that at altitudes of 2,000 m and 5,000 m, the partial pressure of oxygen during inspiration is decreased to 25 and 50% of that at sea level, respectively (7). When exposed to altitudes exceeding 2,500 m, the physiological parameters of the human body, such as blood oxygen saturation, respiratory rate, and heart rate, undergo dramatical changes. A few unacclimatized individuals May also suffer from high-altitude illnesses (HAI), including acute mountain sickness (AMS), high altitude pulmonary edema (HAPE), or high-altitude cerebral edema (HACE), due to the effects of hypobaric hypoxia exposure (4, 8, 9). Consequently, the detection of cerebral oxygen saturation is of utmost importance for both individuals who are acutely exposed to high altitudes and those who are resident in such areas. Early recognition and prompt intervention in cases of decreased rSO2 can effectively avoid adverse outcomes and improve the quality of life at high altitudes. Near-infrared spectroscopy (NIRS) is a promising optical neuroimaging technique, noted for its non-invasiveness, cost-effectiveness, portability, and wearability, making it particular suitable for the longitudinal and continuous monitoring the hemodynamic changes (10, 11). Regional cerebral oxygen saturation (rSO2) reflects the balance between cerebral oxygen supply and consumption within a target brain area. NIRS-based cerebral oximeter has been approved by the US Food and Drug Administration (FDA) for non-invasive and real-time monitoring of rSO2. In recent years, NIRS-based cerebral oximeters have been increasingly utilized as a valuable tool in monitoring rSO2 in various clinical applications, including cardiac surgery, anesthesia, intensive care, pediatric brain injury, and sports medicine (12–16), providing critical insights into brain function and metabolism and aiding the formulation of individualized medical interventions.

In the past few years, several studies have been conducted to explore the physiological functions under high-altitude environment. For example, Saito et al. (17) investigated the impact of workload on cardiovascular parameters and rSO2 in untrained trekkers at altitudes of 2,700 m and 3,700 m, and reported that the resting values of rSO2 and HR did not exhibit significant differences between sea level and the altitudes of 2,700 m and 3,700. However, a dramatic decline in rSO2 after exercise was observed at higher altitudes (approximately 3,000 m), suggesting that an acute decrease in regional cerebral oxygen saturation May could be a primary factor of headache or acute mountain sickness among unacclimatized trekkers (17). A prospective observational cohort study explored the relationship between AMS and physiological health during a 19-day high-altitude expedition reaching 5,372 m. The results showed that an increased heart rate (HR), reduced arterial oxygen saturation (SpO2), and upper respiratory symptoms are either causally linked to AMS or share a common underlying mechanism (18). Xing et al. (19), investigated the effects of race and acclimatization on resting hemodynamics at an altitude of 3,658 m. The results demonstrated that Tibetans exhibit a unique cerebral hemodynamic adaptation to hypobaric hypoxia (HH) exposure when compared to Han immigrants and Han newcomers at high altitude (19). Croker et al. (20) investigated the effects of high altitude on peripheral oxygen saturation (SpO2) and respiratory rate (RR) values in heathy children. The study concluded that diagnostic thresholds for pneumonia should be adjusted based on varying altitudes, as SpO2 levels decrease and respiratory rates increase with rising elevation. The study underscores the significant impact of high altitude on the physiological characteristics of children residing at different altitudes. Zhong et al. (9) explored the potential benefits of remote ischemic preconditioning (RIPC) during hypobaric hypoxia (HH) exposure in a hypobaric chamber simulating 4,000 m. The findings suggested that RIPC intervention improves regional cerebral oxygenation compared to the sham condition, indicating that RIPC May serve as a useful strategy to facilitate acclimatization to high altitude (9).

A variety of studies have demonstrated the benefits of oxygenation monitoring in high-altitude areas for evaluating the balance of oxygen supply and oxygen demand. However, it is noteworthy that several studies were conducted under hypobaric chamber settings, which May not accurately reflect the complexities of real high-altitude environment. Moreover, the majority of these studies have focused on peripheral oxygen saturation (SpO2), with a relative scarcity of research concentrated on regional cerebral oxygen saturation (rSO2). The possible reasons for this discrepancy include: (1) The invasive nature of jugular venous blood saturation (SjO2) detection limits the suitability for widespread use; (2) Although pulse oximeters are commonly used for oxygen detection at high altitudes, they only provide readings of peripheral arterial oxygen saturation (SpO2), which cannot accurately reflect the cerebral oxygen saturation (rSO2); (3) Traditional NIRS-based oximeters, which typically comprise a control unit, multiple optodes, and connecting cables or fibers, and often necessitate the operation by medical professionals, leading to a shortage of wireless, wearable oximeters suitable for cerebral oxygenation detection in particular situations such as sport science, pre-hospital care, and high-altitude environments. In high-altitude related diseases, symptoms affecting the brain or nervous system are frequently pronounced and pose a significant risk Timely intervention is crucial, as delayed action can impact the overall prognosis. Therefore, rSO2 monitoring could offer a more direct and potentially valuable approach for clinical research, particularly in assessing the impact of high-altitude conditions on the brain’s oxygen status.

NIRS technology has demonstrated its efficacy as a useful and promising instrument for the detection of cerebral oxygen saturation under the demanding conditions encountered in extreme high-altitude environments. It provides a sensitive and reliable method for measuring rSO2. The advent of wearable, wireless cerebral oxygen oximeters (WORTH band), which are characterized by their portability and user-friendly simple operation, has significantly enhanced the ability to conduct facilitated the real-time and continuous monitoring of rSO2 in high altitudes, providing a valuable tool for future research in this field.

In this study, simultaneous acquisition of physiological characteristics, including regional cerebral/tissue oxygen saturation and heart rate was conducted during exposure to various levels of high altitudes. The primary objective of this research was to elucidate the acute alternations in physiological parameters for lowlanders newly arrived at different high altitudes. A secondary objective was to investigate the fluctuations in rSO2 among elderly individuals engaging in high-altitude cycling. We hope that the findings of this study could expand the current knowledge of high altitude-induced hypoxia and contribute to the enhancement of medical care for the human brain under such conditions.

## 2 Materials and methods

### 2.1 High-altitude expedition

A total of 24 healthy adult males (aged between 18 and 23 years) from low-altitude areas participated the Tibet high-altitude expedition research. The exclusion criteria included: (1) Subjects with a history of neurological or psychiatric disorders, including depression, cardiovascular, and respiratory diseases, were not eligible to participate; (2) Subjects with a record of medication use within the preceding 2 months were excluded to avoid penitential confounding effects; (3) Subjects already residing at high-altitude areas (above 2,500 m) or those who had previously traveled to such areas were not considered for this study to maintain a uniform baseline for acclimatization responses. Written informed consent was obtained from each participant. The current study was approved by the ethics committee of the Seventh Medical Center of Chinese PLA General Hospital.

**Figure 1:**
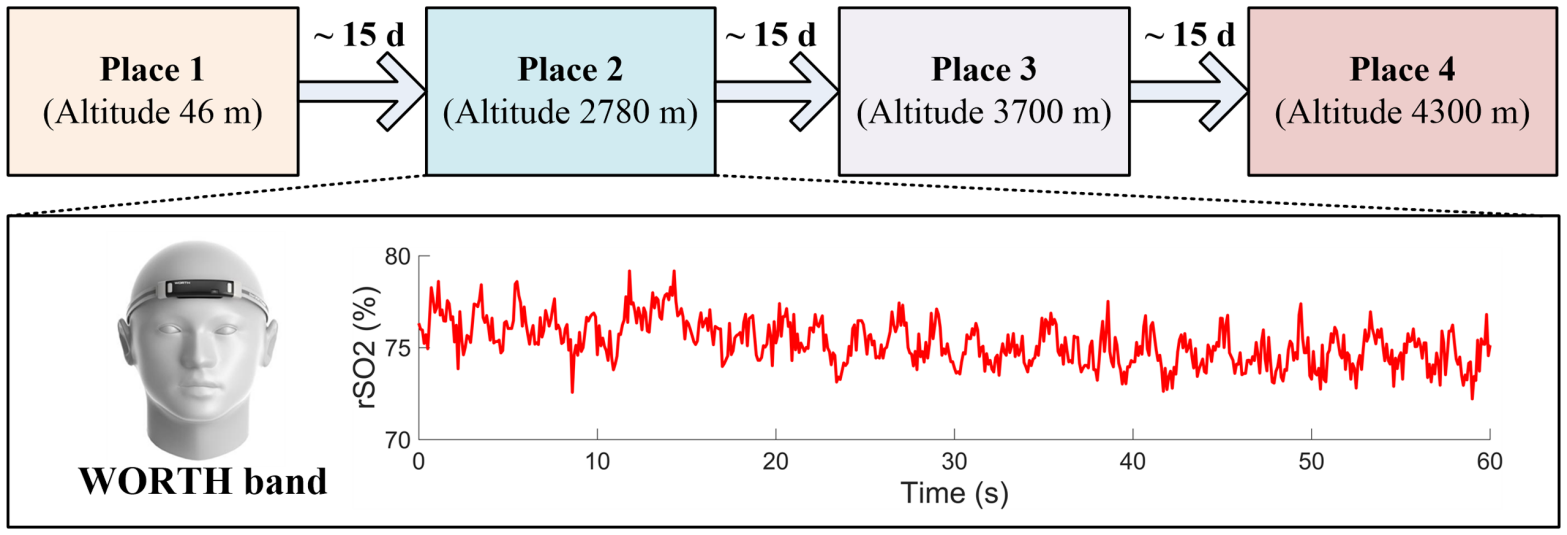
The experimental paradigm for the high-altitude expedition.

The participants were required to complete a two-month expedition, which encompassed a baseline period at low altitude (approximately 46 m), followed by a gradual ascent to three different places, each at incrementally higher altitudes of 2,780 m, 3,700 m, and 4,300 m. Given the cyclical nature of breathing patterns and cardiac function, it’s essential to measure the rSO2 over a few seconds to derive a reliable average data, rather than relying on a single-point visual check (21). Therefore, in this study, the physiological characteristics, including cerebral oxygen saturation (rSO2) and heart rate (HR) values, were continuously recorded over 60 s using a wearable wireless cerebral oximeter (WORTH band; Casibrain Technology, China) (16), so as to ensure the collection of robust and accurate rSO2. In the WORTH band oximeter, a light emitting diode (LED) source with two wavelengths, 760 nm and 840 nm were used. The sampling rate of the system was 10 Hz. Specifically, clinical assessments were conducted before the expedition, with simultaneous recordings of the regional cerebral oxygen saturation (rSO2) and heart rate taken to establish a baseline for the participants at an altitude of 46 m. After that, the rSO2 and heart rate values were continuously acquired at three incremental altitudes (2,780 m, 3,700 m, and 4,300 m) within 24 h upon arrival at each altitude. This protocol was designed to capture the acute changes in rSO2 levels that occur in response to the rapid change in altitude. To facilitate acclimatization and reduce the risk of high altitude-induced acute mountain sickness (AMS), participants were allowed to stay at each area for approximately 15 days. In this study, owing to the challenges posed by high-altitude acute mountain sickness and other factors, 5 subjects were unable to complete the entire expedition. Consequently, the data from the remaining 19 participants were used for further analysis. The experimental paradigm for the high-altitude expedition is illustrated in Figure 1.

### 2.2 High-altitude cycling in elderly subjects

Two elderly participants (one 68-year-old male and one 67-year-old male) were recruited for this study. They were in good health and had no reported history of neurological or psychiatric disorders. They were low-altitude (approximately 400 m above sea level in Shanxi, China) natives and had no prior experience with high-altitude (above 2,500 m) exposure. Written informed consent was obtained from each participant. The current study was approved by the ethics committee of the Seventh Medical Center of Chinese PLA General Hospital.

**Figure 2:**
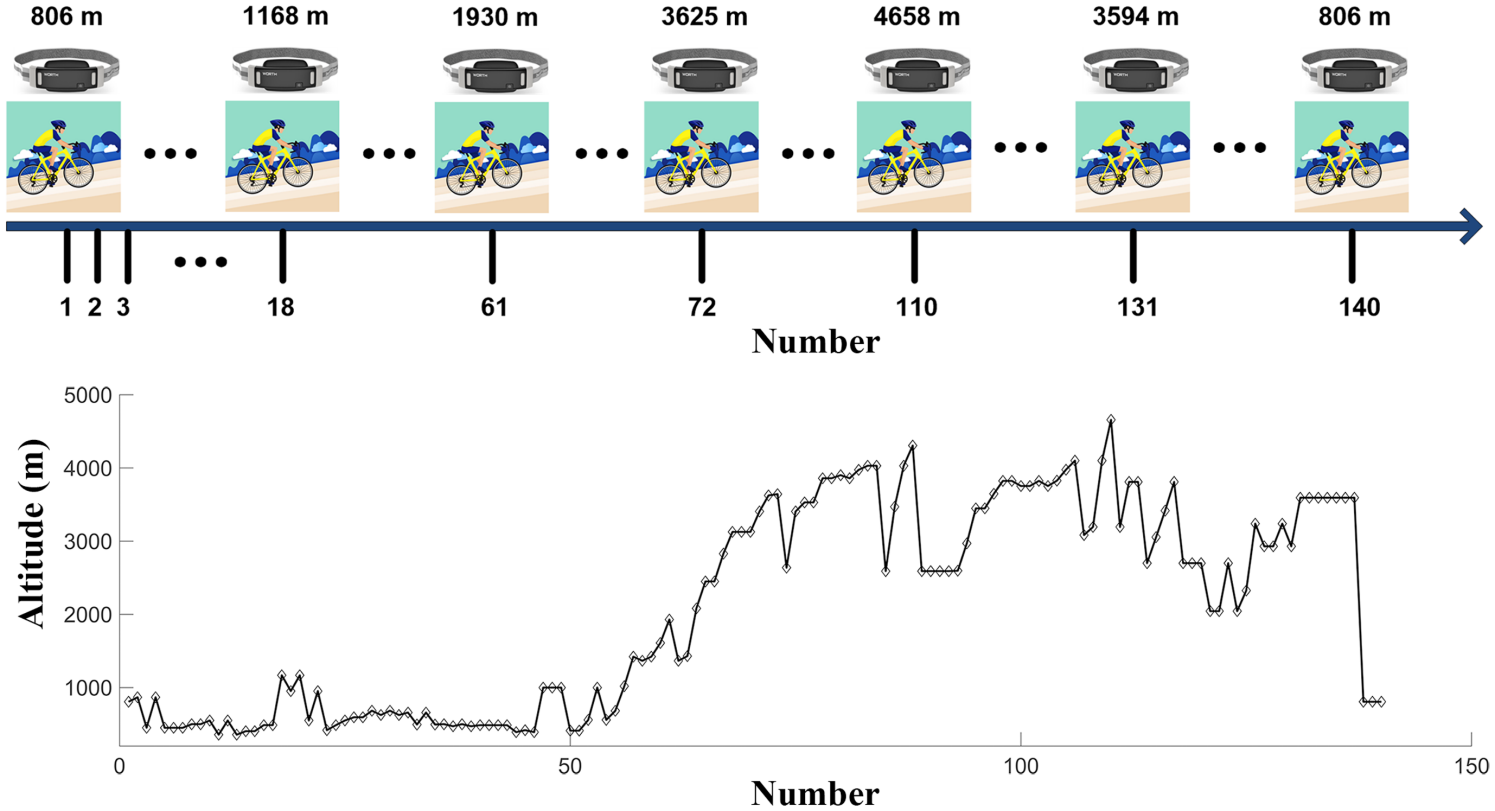
The experimental paradigm for the high-altitude cycling. The numbers indicate the serial order for the measurement of physiological features at different altitudes.

This study involved a high-altitude cycling experiment that lasted for 52 days and covered a range of altitudes from 356 m to 4,658 m across different altitudes in China. During the experiment, the regional cerebral oxygen saturation (rSO2) and heart rate (HR) were acquired using a wearable wireless cerebral oximeter (WORTH band; Casibrain Technology, China) (16). Additionally, the peripheral oxygen saturation (SpO2) was monitored using a non-invasive pulse oximeter (YX301, Yuwell Medical Systems). Specifically, the rSO2, SpO2 and heart rate values were acquired more than twice a day to record the real-time physiological data, totally yielding 140 measurements. Subsequently, to explore the physiological adaptations required for high-altitude cycling in the elderly subjects, the relationship between these physiological parameters and the varying altitudes was analyzed using Pearson correlation. The experimental paradigm for the high-altitude cycling in the elderly subjects is illustrated in Figure 2.

## 3 Data analysis

In this study, the oxygen saturation data were analyzed using MATLAB 2019a platform (MathWorks Inc., Natick, Massachusetts, United States). Specifically, the raw oxygen saturation data were firstly filtered with spline interpolation method to remove the large motion artifacts. Then, rSO2 mean value was calculated from 60 s rSO2 data for further comparison.

Statistical analysis was performed using the SPSS platform (version 18, SPSS Inc., Chicago, Illinois). The differences between different conditions were quantitatively compared using one-way analyses of variance (ANOVAs). For *post hoc* analysis, the least-significant difference (LSD) and/or Bonferroni’s correction was conducted when appropriate. Pearson correlation was performed to investigate the relationship between different variables. The results were considered statistically significant at *p* < 0.05. In the current study, the results are showed as means ± standard error (SE), unless otherwise mentioned.

## 4 Results

### 4.1 The results of the high-altitude expedition

**Figure 3:**
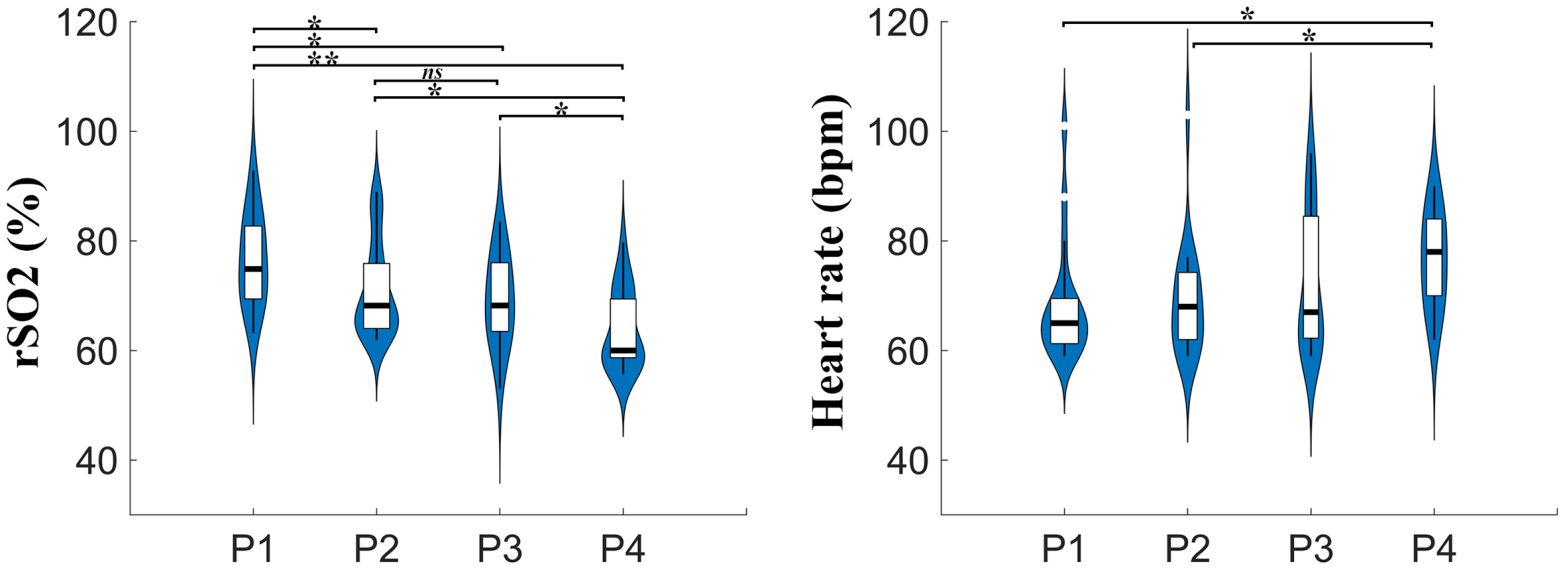
The group-averaged results of rSO2 and heart rate values at four places with different altitudes. rSO2, regional cerebral oxygen saturation; bpm, beats per minute; P1, P2, P3, P4 indicate places with different altitudes 46 m, 2,780 m, 3,700 m, and 4,300 m, respectively.

In this study, the physiological characteristics, including regional cerebral oxygen saturation and heart rate were acquired simultaneously at different high altitudes. The group-averaged results of rSO2 and heart rate in four places with different altitudes are shown in Figure 3.

Quantitatively, the differences of rSO2 and heart rate at different altitudes were compared. As expected, rSO2 exhibited a significant decline with increasing altitudes at 2,780 m, 3,700 m, and 4,300 m when compared to baseline at 46 m. Specifically, the ANOVA results confirmed that the rSO2 values across the four places with different altitudes were significantly different from each other [*F*(3, 72) = 8.202, *p* < 0.001]. Further analysis revealed that the group-averaged rSO2 values at the baseline place, P1 (76.49 ± 1.85%), were significantly larger than those at P2 (70.94 ± 1.97%, *p* = 0.034 LSD corrected), P3 (69.66 ± 1.81%, *p* = 0.005 LSD corrected; *p* = 0.032 Bonferroni corrected), and P4 (63.86 ± 1.63%, *p* < 0.001 Bonferroni corrected), respectively. In addition, the rSO2 values at P2 were found to be significantly greater than those at P4 (*p* = 0.008 LSD corrected; *p* = 0.045 Bonferroni corrected). Similarly, the difference in rSO2 between P3 and P4 achieved statistical significance (*p* = 0.045 LSD corrected). The significant differences in rSO2 across various altitudes highlight the importance of monitoring cerebral oxygen saturation in high-altitude settings, particularly for subjects new to such high-altitude conditions.

During the incremental ascent to high-altitude areas, the difference of the heart rate (HR) values among the four places with different altitudes did not reached statistical significance [*F*(3, 72) = 2.368, *p* = 0.078] at the group level. Notably, the HR values at the highest altitude, P4 (76.47 ± 1.92 bpm), were significantly larger than those at P1 (68.32 ± 2.46 bpm, *p* = 0.02 LSD corrected) and P2 (69.05 ± 2.35 bpm, *p* = 0.034 LSD corrected). However, the difference in HR values between P3 (72.53 ± 2.88 bpm) and P4 was not significant.

### 4.2 The results of the high-altitude cycling in elderly subjects

**Figure 4:**
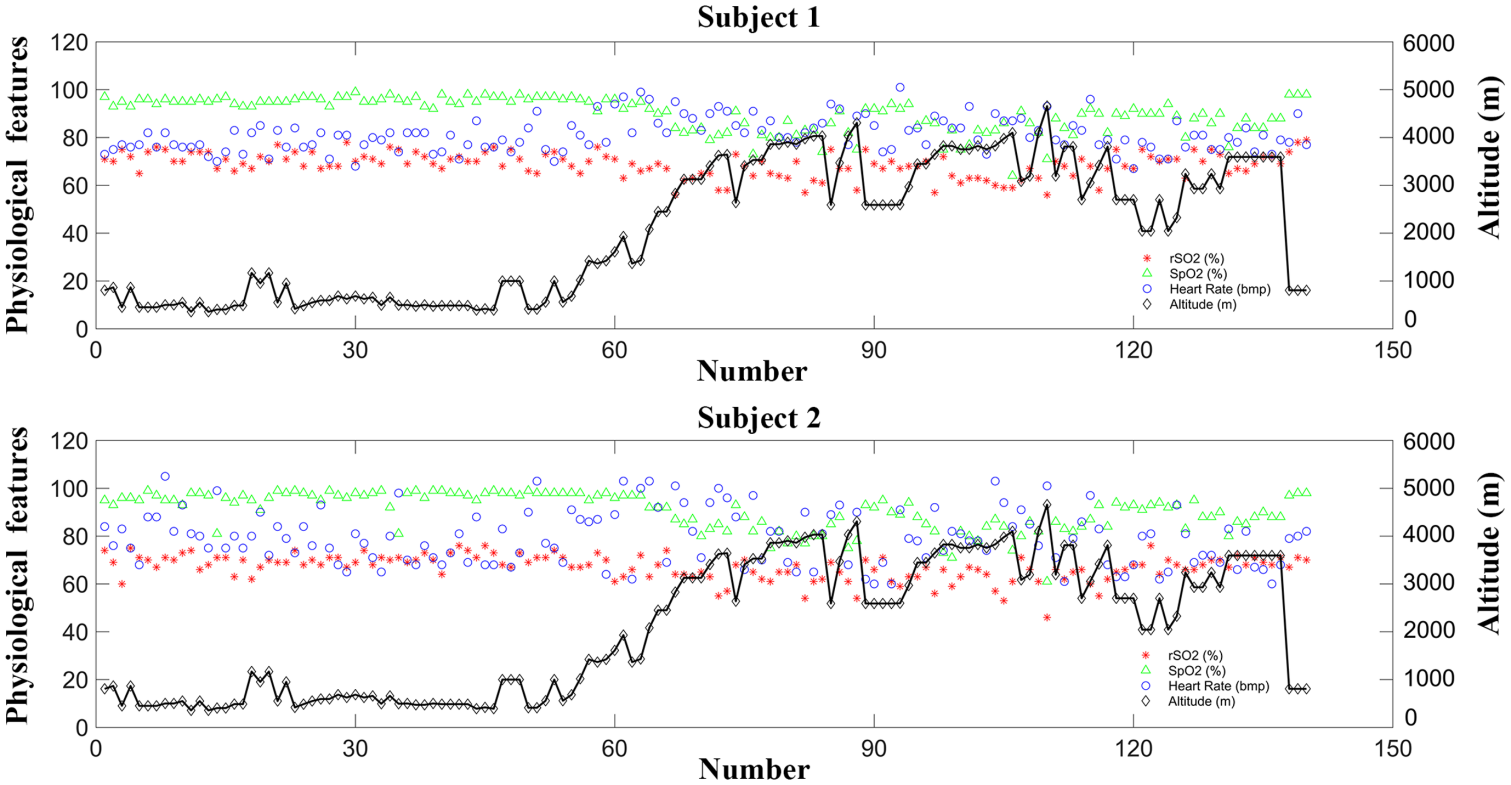
Changes of the physiological features (rSO2, SpO2, and heart rate) across different altitudes for the two elderly subjects.

**Figure 5:**
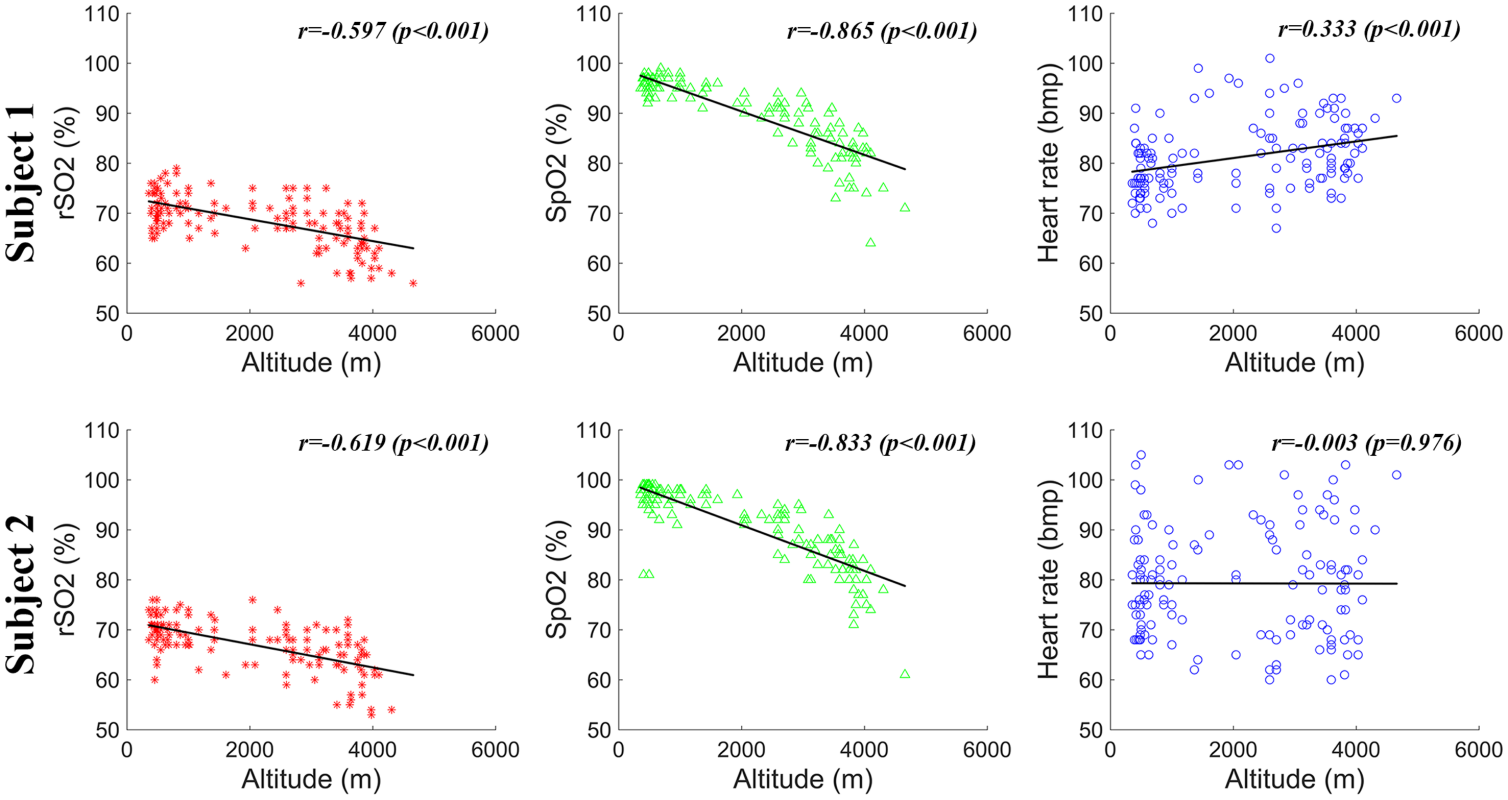
Correlation results between different physiological features (rSO2, SpO2, and heart rate) and the corresponding altitudes for the two elderly subjects.

In this study, the physiological characteristics, including rSO2, SpO2, and heart rate, were measured at various altitudes during the high-altitude cycling and are sequentially displayed in Figure 4. The correlation results between the physiological features and the corresponding altitudes are illustrated in Figure 5.

As shown in Figures 4, 5, the rSO2 and SpO2 values were relatively stable at the altitudes below 2,500 m for both subjects. However, a pronounced decline in both rSO2 and SpO2 was observed at incremental altitudes (above 2,500 m) when compared to the baseline measurements for each elderly subject. Additionally, there was an increase in the heart rate values with the rising altitudes. It is noteworthy that, in comparison to the rSO2 and SpO2 data, the heart rate values exhibited poor stability, with greater fluctuations, especially for Subject 2.

Quantitative analysis was conducted to further investigate the relationship between the physiological parameters and the corresponding altitudes during the high-altitude cycling using Pearson correlation. Strong negative correlations were found between the altitudes and the physiological features (rSO2 and SpO2). Specifically, for Subject 1, a significant correlation was observed between altitudes and rSO2 (*r* = −0.597, *p* < 0.001), as well as between altitudes and SpO2 (*r* = −0.865, *p* < 0.001) values. The findings indicate that as altitude increase, rSO2 and SpO2 decrease correspondingly. The same trend was observed for Subject 2, with significant correlations between altitudes and rSO2 (*r* = −0.619, *p* < 0.001), and altitudes and SpO2 (*r* = −0.833, *p* < 0.001). Additionally, the relationship between the altitudes and hear rate (HR) was also explored. For Subject 1, there was a strong positive correlation between altitudes and HR values (*r* = 0.333, *p* < 0.001). However, for Subject 2, the correlation between altitudes and the HR was not statistically significant (*r* = −0.003, *p* = 0.976). These findings underscore the complex physiological adjustments the body undergoes in response to high-altitude environments and the variability in individual responses to these conditions.

## 5 Discussion

### 5.1 The importance the potential of wireless wearable oximeter for oxygenation detection at high altitudes

In recent years, the increased popularity of high-altitude adventures and the expansion of transportation networks have led to a consequent increase in the number of individuals traveling to high-altitude regions. However, the physiological challenges posed by high altitudes, including decreased humidity, lower air temperature, increased ultraviolet radiation, and the challenges of hypobaric hypoxic environments, can significantly impact the human body’s physiological functions (1). Accurate detection of regional cerebral oxygen saturation (rSO2) becomes particularly crucial for the prevention of brain injury in individuals, especially elderly subjects, exposed to high altitudes. For safety, therefore, it is imperative to investigate the brain functional activity at high-altitude areas. Additionally, there is a unique necessity for medical equipment for the detection, prevention, and treatment of high altitude illnesses (HAIs) (4).

The measurement of jugular venous blood saturation (SjvO2) allows the measurement of global oxygen saturation of the brain. However, the invasive procedure of catheter placement can potentially lead to adverse events, such as carotid artery puncture, formation of hematomas, infection, thrombosis, etc. (2). Over the past few years, pulse oximeters gained widespread acceptance in clinical settings; however, they are limited to the measurement of peripheral arterial oxygen saturation (SpO2) and necessitate pulsatile blood flow for accurate readings (16, 22). Notably, in recent years, cerebral oximeters based on NIRS technique have been used for the detection of regional cerebral oxygen saturation (rSO2), providing valuable insights into the balance between the cerebral oxygen supply and demand. Given their unique strengths, NIRS-based oximeters have been approved by the US Food and Drug Administration (FDA) for detecting cerebral and tissue oxygen saturation in numerous clinical applications, including cardiac surgery, anesthesia, neonatology, and sports, etc. Currently, several commercially available cerebral oximeters are utilized for the measurement of rSO2, including the FORE-SIGHT^™^ (CAS Medical Systems, Branford, CN, United States) system, the INVOS^™^ (Covidien, Inc., Boulder, CO, United States) system, NIRO-200NX (Hamamatsu Photonics, Hamamatsu City, Japan) system, etc. However, these systems typically consist of a control unit, several optodes, and associated fiber-optic cables or wires for data transmission. As a results, this kind of design is primarily suited for use as bedside monitors. There is still a lack of a wireless device that could expand cerebral oxygenation monitoring to tight spaces, such as emergency carts or an ambulance, or for use in sports science (16).

To further improve the applicability and expand new clinical applications, a NIRS-based cerebral oximeter, WORTH band, was designed (16). This innovative device offers noninvasive, cost-effective, and reproducible measurement of cerebral oxygen saturation. Its wireless and wearable design affords unique convenience for continuous monitoring of cerebral oxygen saturation in confined environments, such as emergency transport carts, ambulances, and hyperbaric oxygen chamber, as well as in the field of sports science, especially for high-altitude sports like expedition and cycling. The current findings in this study provide a substantial foundation for future research into investigating the cerebral hemodynamics at high altitudes in a variety of subjects, including common healthy subjects, well-trained athletes, and even patients suffering from cardiovascular, respiratory, and metabolic disorders.

### 5.2 Changes of physiological characteristics in high-altitude expedition

In this high-altitude expedition task, quantitative comparisons were made between different altitudes for physiological characteristics, specifically rSO2 and HR. Significant differences were observed in rSO2 values across four different altitudes (46, 2,780, 3,700, and 4,300 m). Notably, the rSO2 demonstrated a significant decrease with increasing altitude at the group level during the high-altitude expedition study. The rSO2 readings at higher altitudes (2,780, 3,700, and 4,300 m) were significantly lower than those recorded at sea level (46 m). Furthermore, a significant drop in rSO2 was observed at the highest altitude (4,300 m) compared to the moderate altitude (2,780 m). However, no significant differences in rSO2 were observed between the altitudes of 2,780 m and 3,700 m, potentially due to the acclimatization effects of chronic exposure. As for the HR, significant differences were observed only in the comparisons between the pairs of (4,300 m vs. 46 m) and (4,300 m vs. 2,780 m) at the group level. These findings suggested that the rSO2 is a more sensitive and effective indicator than HR for evaluating changes in physiological characteristics at high altitudes. This underscores the utility of rSO2 as a key measure for assessing cerebral oxygenation levels and the body’s adaptation to high-altitude environments.

Our results are in concordance with the previous studies. For example, Hadolt and colleagues measured the regional cerebral (rSO2) and peripheral (SpO2) oxygen saturation during a 22-day high-altitude trekking in the Nepal Himalayas (2,850–5,600 m) (7). They found that the there was a significant decrease in both rSO2 and SpO2 at high altitudes, with the decline in rSO2 being more pronounced at higher altitudes compared to SpO2. A previous study investigated the impact of ethnicity and acclimatization to high altitude (3,658 m) on physiological characteristics (19). The results revealed that after O2-inhalation, the arterial oxygen saturation (SaO2) changed from 88.8 ± 0.7% to 98.4 ± 0.2% for Tibetans with lifelong exposure to high altitude. For lowlanders with three-day exposure, SaO2 increased from 85.7 ± 0.9% to 98.6 ± 0.1%, and for lowlander with five-year exposure, SaO2 increased from 90.6 ± 0.3% to 98.7 ± 0.2%, respectively. The results indicate that Tibetans exhibit a unique cerebral hemodynamic regulatory pattern to maintain a high level of stability of oxygen delivery. Additionally, it was reported that both rSO2 and SpO2 were significantly lower, while the microvascular total vessel density (TVD) was higher in newborns born at high altitude (3,840 m) compared to those born at sea level. This phenomenon reflects a general adaptive mechanism that newborns employ to cope with the reduced oxygen availability in high-altitude environments (23).

It has been reported that the human body’s physiological adaptations to hypoxia mainly include cardiovascular, metabolic, respiratory, hemodynamic, and endocrine responses (1). Acute exposure to high altitude stimulates the adrenergic system, leading to decreased regional oxygen saturation, increased heart rate, and elevated cardiac output. Additionally, despite the maintenance of stable blood pressure, there is an increase in pulmonary artery pressure due to hypoxic pulmonary vasoconstriction. Since oxygen molecules diffuse across the alveolar-capillary membrane driven by a pressure gradient, and as the partial pressure of oxygen decreases with increasing altitude, the number of oxygen molecules traveling across the alveolar-capillary membrane is consequently affected by altitude (7). After several days of exposure to high altitudes, the autonomic nervous system undergoes adaptation, leading to a reduction in tachycardia and protecting the myocardium from excessive energy demand. However, permanent exposure to high-altitude areas could evoke erythropoiesis, which, if excessive, even result in chronic high-altitude illnesses (1). Acute exposure to high altitude can lead to adverse effects in patients with cardiovascular disease (1, 4). Nonetheless, intermittent and moderate hypoxia acclimatization May offer therapeutic benefits in the management of certain cardiovascular diseases, including coronary heart diseases and heart failure (1).

In a study simulating oxygen system failure during high-altitude (9,144 m) high-opening (HAHO) parachute jump (24), arterial oxygen saturation (SaO2) data were measured via blood gas analysis; Additionally, cerebral (rSO2), forearm tissue (StO2), and peripheral (SpO2) oxygen saturation values were acquired noninvasively. The quantitative results revealed that rSO2 correlates more closely with SaO2 and can serve as a valuable tool for the evaluation of hypoxemia or hypobaric chamber training. It has been reported that remote ischemic preconditioning (RIPC) can enhance aerobic performance during acute hypobaric hypoxia exposure by accelerating regional oxygenation and enhancing cardiac function, suggesting that RIPC May offer benefits for individuals undergoing acute hypobaric hypoxia exposure (9). These findings highlight the importance of acclimatization for individuals from lowland areas who are venturing into high-altitude environments.

### 5.3 Changes of physiological characteristics in high-altitude cycling of elderly subjects

Recently, there has been a progressive increase in the number of elderly people who travel to high-altitude regions (4). It was reported that approximately 10% of trekkers in Nepal were 50 years or older in 1989 (25). An epidemiologic study conducted in 2016 revealed a pronounced increase, with about 62% of 670 Himalayan trekkers being over 50 years of age (26). Given the potential for increased vulnerability to high-altitude illnesses and the physiological changes associated with aging, real-time monitoring of cerebral oxygenation is of particular importance for elderly individuals at high altitudes. This practice is crucial for ensuring their safety and well-being, allowing for timely interventions that can prevent acute mountain sickness (AMS) and other high-altitude-related complications.

Cerebral oxygenation is closely related to cerebral blood flow, oxygen consumption, and arterial oxygen content. Our wireless, wearable cerebral oximeter (WORTH band) holds the potential to become a useful tool for evaluating cerebrovascular acclimatization during incremental ascent to high altitude. In this study, the regional cerebral (rSO2), peripheral (SpO2) oxygen saturation, and heart rate (HR) of the elderly people were monitored simultaneously during a long-term (52 days) high altitude cycling (from 356 m to 4,658 m) in China. This study centered on exploring the effects of gradual high-altitude exposure on elderly individuals engaged in cycling activities. The research aim was to understand how the distinct physiological characteristics, adapts to and copes with the challenges of high-altitude environments. In this study, observations included a reduction in regional cerebral oxygen saturation (rSO2) and arterial oxygen saturation (SpO2), as accompanied by an increase in heart rate (HR) as altitude increased. Specifically, the rSO2 and SpO2 data were relatively stable at altitudes lower than 2,500 m. However, a dramatic decline in both rSO2 and SpO2 was noted at altitudes (above 2,500 m) when compared to the baseline level for the two subjects. Additionally, the heart rate values were observed to increase with rising altitudes. Notably, when compared to the rSO2 and SpO2 data, the heart rate values exhibited greater fluctuations and presented poorer stability, particularly in Subject 2. The possible explanation for this discrepancy May be attributed to the individual differences in ventilatory and other physiological responses to hypoxia among individuals, suggesting intersubject variability.

At high-altitude regions, the principal physiological systems impacted include the brain, heart, lungs, kidneys, and blood (8). Most physiological responses exhibit a dose–response relationship, with greater degrees of hypoxia triggers larger responses. For example, cerebral blood flow, heart rate, and ventilation typically increase within several minutes of exposure to increased altitude, while changes in plasma volume and serum erythropoietin concentration generally occur within 1–2 days (4). Despite similar patterns and temporal dynamics of the responses, the magnitude of the responses can vary considerably among different individuals. The findings from this study could further improve our understanding of the impact of high-altitude hypoxia on the cardiovascular system, and further enable the provision of better documented and evidence-based guidance for individuals with cardiovascular diseases who are considering traveling to high-altitude environments. Adequate preparation and awareness of the physiological changes that occur can help mitigate risks and ensure a safer experience at altitude for those with pre-existing heart conditions.

### 5.4 Limitations of this study

One of the limitations of this study is the limited sample size for the elderly participants engaged in high-altitude cycling. In subsequent research efforts, the findings of the current study should be further confirmed using data from multi-center studies with larger sample size. The second limitation pertains to the data collection, which was limited to rSO2, SpO2, and heart rate (HR)measurements. To conduct a more comprehensive investigation into the effects of altitude on the human body, future studies should include additional physiological parameters (such as respiratory rate, blood pressure, etc.) to provide a more comprehensive understanding of the body’s response to high altitudes.

Despite these limitations, this study presents a significant step in the exploration of physiological changes o in subjects with high-altitude exposure. Additionally, the current findings suggested that the NIRS-based wireless wearable oximeter (WORTH band), can serve as a valuable instrument for evaluating cerebral oxygenation and detecting potential brain damage at high altitudes, which in turn facilitates physiological monitor-guided, goal-directed management of cerebral oxygenation in high-altitude settings, thereby improving the safety and efficacy of high-altitude activities and medical interventions.

## Data Availability

The raw data supporting the conclusions of this article will be made available by the authors, without undue reservation.

## References

[1] Richalet JP Hermand E. Cardiovascular physiology and pathophysiology at high altitude. Nat Rev Cardiol. (2024) 21:75–88. doi: 10.1038/s41569-023-00924-9 37783743

[2] Rahul J. S. Kakkar G. Cerebral blood flow monitoring. In: Principles and Practice of Neurocritical Care. eds. Prabhakar H. Singhal V. Zirpe K. G. Sapra H.. Singapore: Springer, (2024). doi: 10.1007/978-981-99-8059-8_6

[3] Quindry J Dumke C Slivka D Ruby B. Impact of extreme exercise at high altitude on oxidative stress in humans. J Physiol. (2016) 594:5093–104. doi: 10.1113/JP270651 26453842 PMC5023697

[4] Luks AM Hackett PH. Medical conditions and high-altitude travel. N Engl J Med. (2022) 386:364–73. doi: 10.1056/NEJMra2104829 35081281

[5] Lafave HC Zouboules SM James MA Purdy GM Rees JL Steinback CD. Steady-state cerebral blood flow regulation at altitude: Interaction between oxygen and carbon dioxide. Eur J Appl Physiol. (2019) 119:2529–44. doi: 10.1007/s00421-019-04206-6 31559499

[6] Ze B Liu L Yang Jin GS Shan M Geng Y Zhou C. Near-infrared spectroscopy monitoring of cerebral oxygenation and influencing factors in neonates from high-altitude areas. Neonatology. (2021) 118:348–53. doi: 10.1159/000514403 34107488

[7] Hadolt I Litscher G. Noninvasive assessment of cerebral oxygenation during high altitude trekking in the Nepal Himalayas (2850-5600 m). Neurol Res. (2003) 25:183–8. doi: 10.1179/016164103101201175 12635520

[8] Mallet RT Burtscher J. Molecular mechanisms of high-altitude acclimatization. Int J Mol Sci. (2023) 24:1698. doi: 10.3390/ijms24021698 36675214 PMC9866500

[9] Zhong Z Dong H Wu Y Zhou S Li H Huang P. Remote ischemic preconditioning enhances aerobic performance by accelerating regional oxygenation and improving cardiac function during acute hypobaric hypoxia exposure. Front Physiol. (2022) 13:950086. doi: 10.3389/fphys.2022.950086, PMID: 36160840 PMC9500473

[10] Jobsis FF. Noninvasive, infrared monitoring of cerebral and myocardial oxygen sufficiency and circulatory parameters. Science (New York, NY). (1977) 198:1264–7. doi: 10.1126/science.929199, PMID: 929199

[11] Yücel MA Lühmann AV. Best practices for fNIRS publications. Neurophotonics. (2021) 8:012101. doi: 10.1117/1.NPh.8.1.012101 33442557 PMC7793571

[12] Huppert EL Parnia S. Cerebral oximetry: a developing tool for monitoring cerebral oxygenation during cardiopulmonary resuscitation. Ann N Y Acad Sci. (2022) 1509:12–22. doi: 10.1111/nyas.14706, PMID: 34780070

[13] Koch KU Zhao X Mikkelsen IK Espelund US Aanerud J Rasmussen M. Correlation between cerebral tissue oxygen saturation and oxygen extraction fraction during anesthesia: monitoring cerebral metabolic demand-supply balance during vasopressor administration. J Neurosurg Anesthesiol. (2023) 35:238–42. doi: 10.1097/ANA.0000000000000822, PMID: 34861671

[14] Mulkey SB Polglase GR. Cerebral oxygen saturation-a useful bedside vital sign for neonatal encephalopathy. J Perinatol. (2021) 41:2577–9. doi: 10.1038/s41372-021-00916-y 33547404

[15] Si J Li M Zhang X Han R Ji X Jiang T. Cerebral tissue oximeter suitable for real-time regional oxygen saturation monitoring in multiple clinical settings. Cogn Neurodyn. (2023) 17:563–74. doi: 10.1007/s11571-022-09847-6, PMID: 37265661 PMC10229493

[16] Si J Zhang X Li M Yu J Zhang Z He Q. Wearable wireless real-time cerebral oximeter for measuring regional cerebral oxygen saturation. Science China Inf Sci. (2021) 64. doi: 10.1007/s11432-020-2995-5

[17] Saito S Nishihara F Takazawa T Kanai M Aso C Shiga T. Exercise-induced cerebral deoxygenation among untrained trekkers at moderate altitudes. Arch Environ Health. (1999) 54:271–6. doi: 10.1080/00039899909602485, PMID: 10433186

[18] Oliver SJ Sanders SJ Williams CJ Smith ZA Lloyd-Davies E Roberts R. Physiological and psychological illness symptoms at high altitude and their relationship with acute mountain sickness: a prospective cohort study. J Travel Med. (2012) 19:210–9. doi: 10.1111/j.1708-8305.2012.00609.x, PMID: 22776381

[19] Xing CY Serrador JM Knox A Ren LH Zhao P Wang H. Cerebral blood flow, oxygen delivery, and Pulsatility responses to oxygen inhalation at high altitude: highlanders vs lowlanders. Front Physiol. (2019) 10:61. doi: 10.3389/fphys.2019.00061, PMID: 30792663 PMC6375252

[20] Crocker ME Hossen S Goodman D Simkovich SM Kirby M Thompson LM. Effects of high altitude on respiratory rate and oxygen saturation reference values in healthy infants and children younger than 2 years in four countries: a cross-sectional study. Lancet Glob Health. (2020) 8:e362–73. doi: 10.1016/S2214-109X(19)30543-1, PMID: 32087173 PMC7034060

[21] Tannheimer M Lechner R. The correct measurement of oxygen saturation at high altitude. Sleep Breath. (2019) 23:1101–1106. doi: 10.1007/s11325-019-01784-9 30701422

[22] Sandroni C Parnia S Nolan JP. Cerebral oximetry in cardiac arrest: a potential role but with limitations. Intensive Care Med. (2019) 45:904–6. doi: 10.1007/s00134-019-05572-7 30840118

[23] Gassmann NN van Elteren HA Goos TG Morales CR Rivera-Ch M Martin DS. Pregnancy at high altitude in the Andes leads to increased total vessel density in healthy newborns. J Appl Physiol (1985). (2016) 121:709–15. doi: 10.1152/japplphysiol.00561.2016 27445300 PMC5142254

[24] Ottestad W Kåsin JI Høiseth L. Arterial oxygen saturation, pulse oximetry, and cerebral and tissue oximetry in hypobaric hypoxia. Aerosp Med Hum Perform. (2018) 89:1045–9. doi: 10.3357/AMHP.5173.2018, PMID: 30487024

[25] Shlim DR Houston R. Helicopter rescues and deaths among trekkers in Nepal. JAMA. (1989) 261:1017–9. doi: 10.1001/jama.1989.03420070067032, PMID: 2578027

[26] Keyes LE Mather L Duke C Regmi N Phelan B Pant S. Older age, chronic medical conditions and polypharmacy in Himalayan trekkers in Nepal: an epidemiologic survey and case series. J Travel Med. (2016) 23:taw052. doi: 10.1093/jtm/taw052 27503853

